# Application of Adaptive Robust Kalman Filter Base on MCC for SINS/GPS Integrated Navigation

**DOI:** 10.3390/s23198131

**Published:** 2023-09-28

**Authors:** Linfeng Li, Jian Wang, Zhiming Chen, Teng Yu

**Affiliations:** 1 School of Integrated Circuits and Electronics, Beijing Institute of Technology, Beijing 100081, China; 3420225072@bit.edu.cn (L.L.); czm@bit.edu.cn (Z.C.); 2 Beijing Institute of Electronic System Engineering, Beijing 100854, China; 3 Beijing Institute of Control and Electronic Technology, Beijing 100038, China; wangjianseu@163.com

**Keywords:** adaptive and robust Kalman filter, maximum correntropy criterion (MCC), sliding window method, global positioning system (GPS), strapdown inertial navigation system (SINS)

## Abstract

In this paper, an adaptive and robust Kalman filter algorithm based on the maximum correntropy criterion (MCC) is proposed to solve the problem of integrated navigation accuracy reduction, which is caused by the non-Gaussian noise and time-varying noise of GPS measurement in complex environment. Firstly, the Grubbs criterion was used to remove outliers, which are contained in the GPS measurement. Then, a fixed-length sliding window was used to estimate the decay factor adaptively. Based on the fixed-length sliding window method, the time-varying noises, which are considered in integrated navigation system, are addressed. Moreover, a MCC method is used to suppress the non-Gaussian noises, which are generated with external corruption. Finally, the method, which is proposed in this paper, is verified by the designed simulation and field tests. The results show that the influence of the non-Gaussian noise and time-varying noise of the GPS measurement is detected and isolated by the proposed algorithm, effectively. The navigation accuracy and stability are improved.

## 1. Introduction

The global positioning system (GPS) outputs the velocity and position information of the carrier based on the satellite signals, which contain the characteristics of continuity, real-time, stability, and so on, but its positioning accuracy is easily corrupted by the external environment. Moreover, it cannot output the positioning information without the external satellite signals. Thus, the self-contained characteristic of the GPS is poor [1,2,3,4]. The strapdown inertial navigation system (SINS) is an autonomous navigation system based on Newton’s law of kinematics [5,6]. The three-dimensional attitude, velocity, and position information of the carrier can be obtained by integrating the specific force and angular velocity with inertial navigation algorithm. Its characteristics, which are strong autonomy, anti-interference, and high sampling rate, are the major advantages of the integrated navigation system. But the errors of the SINS accumulate over time, and its stability is poor [7]. Therefore, the integration of the GPS and SINS can effectively make up for the defects of each and improve the stability and reliability of the integrated navigation system [8].

However, in practical applications, the GPS measurements are easily corrupted by the external environment, resulting in measurement noises and outliers, which are no longer obeying Gaussian distribution. Under the assumption of the noises with the Gaussian distribution, the Kalman filter is the optimal unbiased estimation of information fusion. When the measurement noise is no longer obeying Gaussian distribution, the estimation accuracy of the Kalman filter cannot be maintained [9]. To solve this problem, many researchers put forward several methods to improve the performance of the Kalman filter. The Kalman filter based on Huber’s core function, which is based on the l1 and l2 norms, is designed to suppress non-Gaussian noise in [10]. However, these methods still assign some weight to invalid measurements. Using a maximum correlation entropy improvement, the Kalman filter can be improved to address non-Gaussian noises [11,12,13], and the weights of measurement information are updated in real time. The Kalman filter obtains better performance when the non-Gaussian noises are suppressed. Bayesian estimation uses the maximum posterior probability to update the measurement covariance matrix [14], which improves the adaptive characteristics of the Kalman filter. Other methods, which are based on student-t distribution models, are devised in [15], and these methods give the optimal solution with Bayesian estimation, which requires accurate prior information. However, the mixing probability in the time-varying non-Gaussian case does not change fast. At this time, the prior information is no longer reliable.

The above methods deal with one of the non-Gaussian characteristics, and the time-varying characteristics, of the GPS measurement noises, but they fail to deal with both non-Gaussian noise and time-varying noise. Therefore, an adaptive and robust Kalman filter algorithm, which is based on the MCC criterion, is proposed in this paper to suppress non-Gaussian noise and time-varying noise at the same time. Moreover, before giving weight to the different types of measurement information with the MCC criterion, the new outliers in the fixed-length sliding window were detected and eliminated by the Grubbs test method, and the measurement noise covariance matrix was estimated adaptively. In this way, the time variability and non-Gaussian characteristics of measurement noise were processed simultaneously. Moreover, the estimation accuracy of integrated navigation system was improved.

The rest of this paper is organized as follows. Section 2 gives the models of the SINS/GPS integrated navigation system. All of the system equations and the measurement models are shown in detail. In Section 3, the robust Kalman filter, which is based on the MCC criterion, is designed. Moreover, the adaptive filter method, which is based on the adaptive decay factor, is devised with the fixed-length sliding windows method. The simulation and field tests are designed to verify the performance of the proposed method in Section 4. Finally, the conclusions are drawn in Section 5.

## 2. Models of SINS/GPS Integrated Navigation System

In this section, the model of the SINS/GPS integrated system is derived, and the derivations of the model are also investigated. The inertial coordinate frame, which is a non-rotation frame, is defined as *i*-frame. The earth coordinate frame, which is the earth-centered earth-fixed (ECEF) frame, is defined as *e*-frame. The navigation coordinate frame, which is the east–north-up frame, is defined as *n*-frame. The carrier coordinate frame, which is the right–forward–up frame, is defiend as *b*-frame. And the coordinate frame, which is obtained by SINS calculation, is *n*${}^{\prime }$-frame.

### 2.1. The System Model of the SINS/GPS Integration

In this paper, the state vector of SINS/GPS integrated navigation is defined as follows:

(1) $$x={\left[\begin{array}{ccccc} {\left(\varphi \right)}^{⊤} & {\left(v^{n}\right)}^{⊤} & {\left(\delta p\right)}^{⊤} & {\left(\varepsilon^{b}\right)}^{⊤} & {\left(\nabla^{b}\right)}^{⊤} \end{array}\right]}^{⊤}$$

where $\varphi =\left[\begin{array}{ccc} \varphi_{E} & \varphi_{N} & \varphi_{U} \end{array}\right]$ denotes the attitude error between the *n*-frame and calculated *n’*-frame. $\delta v^{n}=\left[\begin{array}{ccc} \delta v_{E} & \delta v_{N} & \delta v_{U} \end{array}\right]$ denotes the velocity error. $\delta p=\left[\begin{array}{ccc} \delta L & \delta \lambda & \delta h \end{array}\right]$ is the position error, being, respectively, the latitude error, longitude error, and height error. $\varepsilon^{b}$ and $\nabla^{b}$ are the dynamic bias of triaxis gyroscopes and triaxis accelerometers.

Based on the aforementioned analysis, the system model of SINS/GPS integrated navigation is expressed as follows:

(2) $$\dot{x}=Fx+w$$

where F denotes the state transition matrix; w denotes the process noise; and F can be calculated by the error equation, which is obtained by the SINS calculation. And the error equation can be expressed as follows:

(3) $$\left\{\begin{array}{cc} \dot{\varphi }= & -\omega_{in}^{n}\times \varphi +\delta \omega_{in}^{n}+C_{b}^{n}\varepsilon^{b}\\ \delta {\dot{v}}^{n}= & C_{b}^{n}f^{b}\times \varphi -\left(2\delta \omega_{ie}^{n}+\delta \omega_{en}^{n}\right)\times v^{n}\\ \; & -\left(2\omega_{ie}^{n}+\omega_{en}^{n}\right)\times \delta v^{n}+C_{b}^{n}\nabla^{b}\\ \delta \dot{L}= & \frac{\delta v_{N}}{R_{M}+h}\\ \delta \dot{\lambda }= & \frac{\delta v_{E}}{R_{N}+h}\sec L+\frac{v_{E}}{R_{N}+h}\sec L\tan L\delta L\\ \delta \dot{h}= & \delta v_{U}\\ {\dot{\varepsilon }}^{b}= & 0\\ \dot{\nabla^{b}}= & 0 \end{array}\right.$$

where $\omega_{in}^{n}$ denotes the angular velocity of the *n*-frame with respect to the *i*-frame expressed in the *n*-frame, and $\delta \omega_{in}^{n}$ is the corresponding error. $\omega_{ie}^{n}$ denotes the angular velocity of the *e*-frame with respect to the *i*-frame expressed in the *n*-frame, and $\delta \omega_{ie}^{n}$ is the corresponding error. $C_{b}^{n}$ denotes the direction cosine matrix (DCM) from the *b*-frame to the *n*-frame. $f^{b}$ denotes the specific force expressed in the *b*-frame. $R_{M}$ is the radius of curvature in meridian, and $R_{N}$ is the radius of curvature in prime vertical.

### 2.2. The Measurement Model of the SINS/GPS Integration

In this paper, the measurement model is defined as the errors of the velocity and position between the SINS and GPS. Thus, the measurement model of the SINS/GPS integrated navigation system can be given as follows:

(4) z=Hx+v

where z is the observable vector. H denotes observable matrix, and v denotes measurement noise.

The observable vector can be expressed as:

(5) $$z=\left[\begin{array}{c} v_{SINS}^{n}-v_{GPS}^{n}\\ p_{SINS}-p_{GPS} \end{array}\right]$$

The observable matrix can be expressed as:

(6) $$H=\left[\begin{array}{ccccc} 0_{3\times 3} & I_{3\times 3} & 0_{3\times 3} & 0_{3\times 3} & 0_{3\times 3}\\ 0_{3\times 3} & 0_{3\times 3} & I_{3\times 3} & 0_{3\times 3} & 0_{3\times 3} \end{array}\right]$$

From the above derivation, it can be found that the accuracy of the integrated system is determined by the external velocity and position, which are obtained from the GPS. However, the positioning accuracy of the GPS is determined by the external circumstances. If the vehicle is moving in the opening area, the positioning accuracy will be well. If the vehicle is moving in the occluded area, the positioning accuracy will be poor. To solve this problem, an adaptive and robust Kalman filter algorithm, which is based on the MCC criterion, is proposed in this paper to suppress non-Gaussian noise and time-varying noise, which are caused by the signal block of the GPS. The derivations of the proposed method are shown in the next sections.

## 3. Adaptive and Robust Kalman Filter Based on MCC

In this section, a robust Kalman filter, which is based on the MCC criterion, is proposed for outliers detection and isolation. Moreover, an adaptive method, which is based on the adaptive decay factor method, is proposed to improve the estimated accuracy of the robust Kalman filter.

### 3.1. Robust Kalman Filter Based on MCC

Correlation entropy is defined as the similarity between two random variables. Define two random variables *X* and *Y*. The correlation entropy between them can be expressed as:

(7) $$V\left(X,Y\right)=E\left[\kappa \left(X,Y\right)\right]=\int \kappa \left(x,y\right)dF_{XY}\left(x,y\right)$$

where E[·] denotes the expectation of the variables, κ(·,·) denotes the kernel function, and $F_{XY}\left(x,y\right)$ denotes the joint probability density function between *X* and *Y*. In this paper, we select the Gaussian kernel as kernel function:

(8) $$\kappa \left(x,y\right)=G_{\sigma }\left(x-y\right)=\exp\left(-\frac{{∥x-y∥}^{2}}{2\sigma^{2}}\right)$$

where σ>0 denotes the kernel bandwidth of correntropy. From Equation (8), we can see that the correntropy of random variable *X* and *Y* with the Gaussian kernel reaches its maximum if and only if X=Y. In practice, the joint probability density is always unknown, so it is common to estimate an approximation of the correlation entropy using the following sampling points:

(9) $$\hat{V}\left(X,Y\right)=\frac{1}{N}\sum_{i=1}^{N}G_{\sigma }\left(x_{i}-y_{i}\right)$$

Then, the covariance of measurement noise is expressed as:

(10) $$E\left[v_{k}v_{k}^{⊤}\right]=\lambda_{k}R_{k}=B_{k}B_{k}^{⊤}$$

where $\lambda_{k}$ is the decay factor.

Left multiplying both sides of Equation (4) by $B_{k}^{-1}$ yields the linear regression model:

(11) $$\xi_{k}=D_{k}-g\left(x_{k}\right)$$

where, $\xi_{k}=B_{k}^{-1}v_{k}$ , $D_{k}=B_{k}^{-1}z_{k}$, $g\left(x_{k}\right)=B_{k}^{-1}H_{k}x_{k}$.

According to the least squares principle and maximum correlation entropy criterion, the cost function is constructed as follows:

(12) $$J_{L}\left(x_{k}\right)={∥x_{k}-{\hat{x}}_{k|k-1}∥}_{{\left(P_{k|k-1}\right)}^{-1}}^{2}+\sum_{i=1}^{m}\rho_{MCC}\left(\xi_{k,i}\right)$$

where ${∥x∥}_{A}^{2}=x^{⊤}Ax$ denotes the quadratic form with respect to A, $x_{k}$ is the actual state at time instant *k*, ${\hat{x}}_{k|k-1}$ is the prediction of state at time instant *k*, $P_{k|k-1}$ represents the corresponding error covariance, $\xi_{k,i}$ is the i−th element of $\xi_{k}$, and *m* refers to the dimension of the measurement vector. The core function $\rho_{MCC}\left(\cdot \right)$ can be given by:

(13) $$\rho_{MCC}\left(\xi_{k,i}\right)=\frac{\left(1-\exp\left(-\xi_{k,i}^{2}/2\sigma^{2}\right)\right)}{\sqrt{2\pi }\sigma }$$

The optimal estimate of the state vector is obtained by minimizing the cost function:

(14) $${\hat{x}}_{k}=\arg\min_{x_{k}}\left({∥x_{k}-{\hat{x}}_{k|k-1}∥}_{{\left(P_{k|k-1}\right)}^{-1}}^{2}+\sum_{i=1}^{m}\rho_{MCC}\left(\xi_{k,i}\right)\right)$$

The optimal solution can thus be found by differencing the cost function with respect to $x_{k}$ as:

(15) $${\left(P_{k|k-1}\right)}^{-1}\left(x_{k}-{\hat{x}}_{k|k-1}\right)-\sum_{i=1}^{m}\psi \left(\xi_{k,i}\right)\frac{\partial \xi_{k,i}}{\partial x_{k}}=0$$

where $\psi \left(e_{k,i}\right)=-G_{\sigma }\left(\xi_{k,i}\right)e_{k,i}$; by defining the function and dialog matrix $C_{k,i}=-\frac{\psi \left(\xi_{k,i}\right)}{\xi_{k,i}}=G_{\sigma }\left(\xi_{k,i}\right)$, Equation (14) can be rewritten as:

(16) $${\left(P_{k|k-1}\right)}^{-1}\left(x_{k}-{\hat{x}}_{k|k-1}\right)-H_{k}^{⊤}B_{k}^{-⊤}C_{k}B_{k}^{-1}\left(z_{k}-H_{k}x_{k}\right)=0$$

Let ${\bar{R}}_{k}=B_{k}C_{k}^{-1}B_{k}^{⊤}$ and ${\hat{x}}_{k|k}=x_{k}$; thus,

(17) $$P_{k|k-1}^{-1}\left({\hat{x}}_{k|k}-{\hat{x}}_{k|k-1}\right)=H_{k}^{⊤}R_{k}^{-1}\left(z_{k}-H_{k}{\hat{x}}_{k|k}\right)$$

Referring to [12] for the equation simplification and derivation process, the measurement update can be given by:

(18) $$\begin{array}{c} \begin{array}{cc} {\hat{x}}_{k|k} & ={\hat{x}}_{k|k-1}+K_{k}\left(z_{k}-H_{k}{\hat{x}}_{k|k-1}\right)\\ K_{k} & =P_{k|k-1}H_{k}^{⊤}{\left(H_{k}P_{k|k-1}H_{k}^{⊤}+{\bar{R}}_{k}\right)}^{-1}\\ P_{k|k} & =\left(I-K_{k}H_{k}\right)P_{k|k-1}{\left(I-K_{k}H_{k}\right)}^{⊤}+K_{k}{\bar{R}}_{k}K_{k}^{⊤} \end{array} \end{array}$$

Using the aforementioned method, the outliers, which are contained in the GPS outputs, can be suppressed. Then, the estimated results will be stable. However, the accuracy of the robust Kalman filter will degrade when there are outliers. To address this problem, an adaptive filter method is investigated in the next subsection.

### 3.2. Adaptive Estimation with the Decay Factor

In order to improve the estimation accuracy of the MCC algorithm when there is time-varying noise, the unknown decay factor $\lambda_{k}$ is necessary to determine in advance. In this paper, the outliers are eliminated with the residual sliding window by the Grubbs test, and then we use two different calculation methods of the new information covariance matrix to estimate the decay factor adaptively. It is noted that the Grubbs test serves to calculate the adaptive decay factor and does not eliminate the measured outliers, so it does not play a direct role in suppressing time-varying noise.

The Grubbs test, also known as the extreme studentized deviate test, is the most effective method to distinguish outliers in the case of normal distribution. It can be described as:

(19) $$G_{i}=\frac{x_{i}-\mu }{\sigma }$$

where $x_{i}$ represents the residual in a fixed-length sliding window, μ represents the mean value, and σ represents the standard deviation. The confidence level was set as 0.95, and the length of the sliding window *M* was determined by the minimization variance parameter [16]. When $G_{i}>G_{p}\left(M\right)$, the data are considered as outliers and removed from the sliding window method to be calculated.

According to the measurement equation, the new information covariance matrix can be expressed as:

(20) $${\hat{P}}_{zz,k|k-1}=H_{k}P_{k|k-1}H_{k}^{⊤}+\lambda_{k}R_{k}$$

where $\lambda_{k}=\mathrm{diag}\left\{\lambda_{1},\lambda_{2},\cdots ,\lambda_{m}\right\}$.

Under the assumption that the residual is stable, the residual covariance matrix ${\hat{P}}_{zz,k|k-1}$ can also be obtained by using the residual $\varepsilon_{k}=z_{k}-H_{k}{\hat{x}}_{k|k-1}$ in the fixed-length sliding window:

(21) $${\hat{P}}_{zz,k|k-1}=\frac{1}{M}\sum_{i=1}^{M}\varepsilon_{k-i}\varepsilon_{k-i}^{⊤}$$

Let $N_{k}={\hat{P}}_{zz,k|k-1}-H_{k}P_{k|k-1}H_{k}^{⊤}$, at this time, decay factor can be expressed as:

(22) $$\lambda_{i}=\frac{\sum_{j=1}^{m}R_{k}\left[i,j\right]N_{k}\left[i,j\right]}{\sum_{j=1}^{m}R_{k}^{2}\left[i,j\right]},\;i=1,2,\cdots ,m$$

Based on the aforementioned method, an adaptive and robust Kalman filtering method for SINS/GPS is devised. In the next section, the simulation and field tests are designed for verifying the performance of the proposed method.

## 4. Simulation and Field Tests

In order to verify the performance of the algorithm, which is proposed in this paper, the comparison tests between simulation data and field tests data are presented respectively, and the position errors of KF [6], VBKF [14], MCCKF [11], and MCCRKF are designed for comparison.

### 4.1. Simulation Test

In the simulation test, a test, which lasts 996 s, is designed for the verification of the SINS/GPS integrated navigation system. It is noted that the errors of the integrated navigation system will converge to a stable value when the filter converges to stable states. Thus, it is not necessary to extend the integrated time. Taking the actual sensor parameters as an example, the simulated parameters of the designed test are shown in Table 1. The initial position of the carrier is set as $108.9^{\circ }$ E, $34.2^{\circ }$ N, and 380 m height; the initial velocity is set as 0 m/s; and the initial attitude angle is set as $0^{\circ }$. The iterations of the VBKF algorithm are set as 3, and the Gauss kernel bandwidth of the MCCKF algorithm and the MCCRKF algorithm is set as 3. Four groups of comparison experiments were set up. Gaussian noise was added to the measurement in test 1, non-Gaussian noise was added to the measurement in test 2, time-varying noise was added to the measurement in test 3, and time-varying non-Gaussian noise was added to the measurement in test 4.

**Figure 1:**
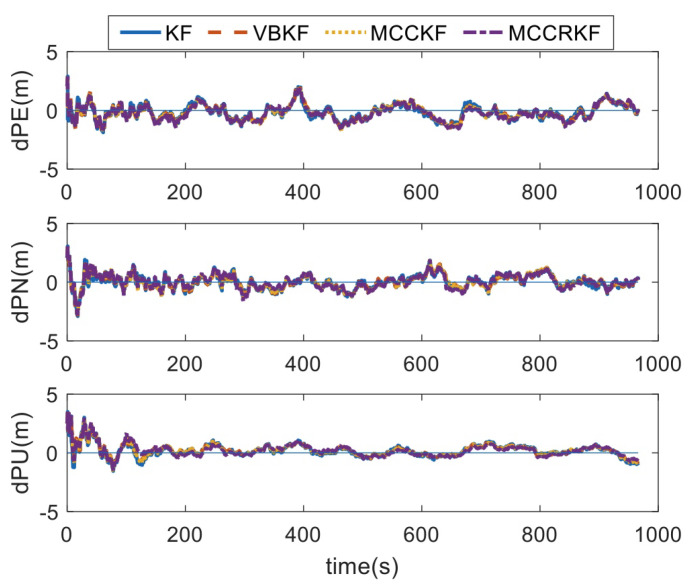
The positioning error of different algorithms under Gaussian noise.

In test 1, the measurement noises of the sensors and the GPS errors are shown in Table 1. Using the designed methods, the positioning errors, which are summarized with the different four algorithms, are shown in Figure 1. With the test results, the RMSE errors of the positioning error are shown in Table 2.

It can be found that, when the measurement noise conforms to Gaussian distribution, the RMSE errors of the positioning errors of the four algorithms are equivalent.

**Figure 2:**
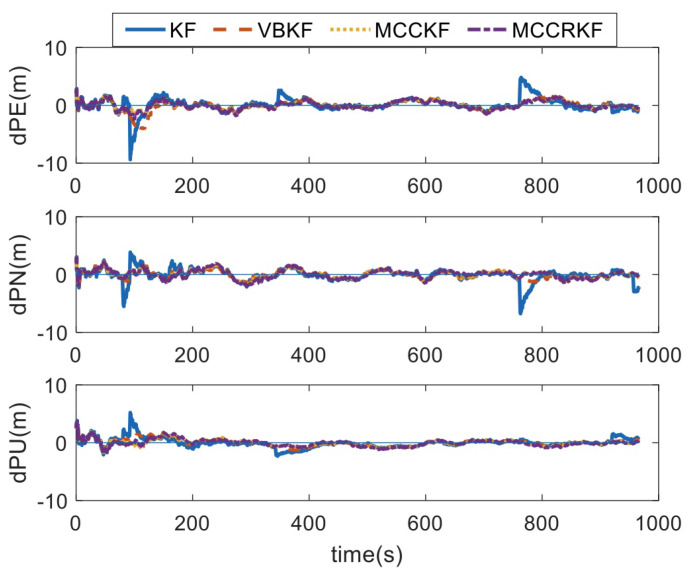
The positioning error of different algorithms under non-Gaussian noise.

The measured noise distribution in test 2 is shown in Formula (23), which represents the heavy-tail distribution. Using these type noises, the positioning errors of the four different algorithms are shown in Figure 2, and the RMSE errors of the positioning error statistical are shown in Table 3.

(23) $$v_{k}∼0.99N\left(0,R_{k}\right)+0.01N\left(0,400R_{k}\right)$$

It can be found that, when the measurement noises are non-Gaussian, the estimated errors of the KF algorithm increase by one time; the estimated errors of the VBKF algorithm also increase significantly; while the estimated errors of MCCKF algorithm and MCCRKF algorithm are equivalent, and their estimated accuracy is consistent with the same one when the measurement noises are Gaussian.

**Figure 3:**
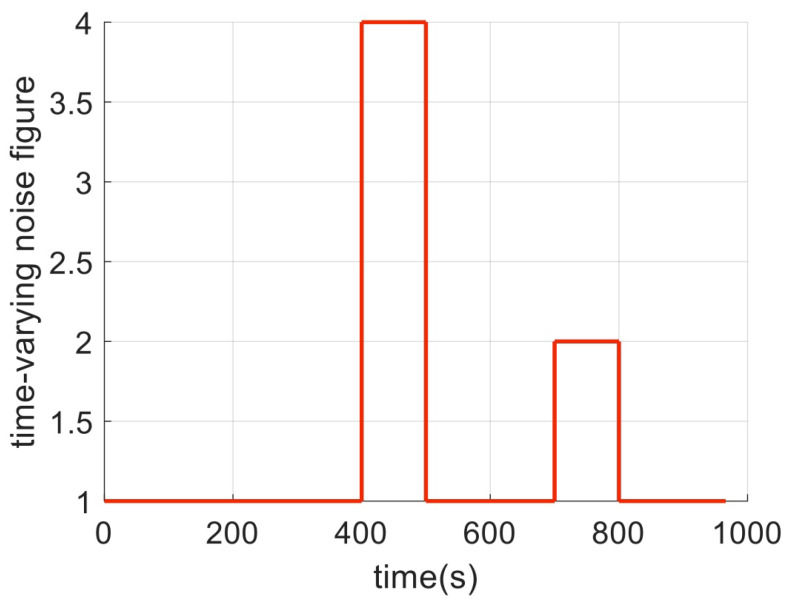
Time-varying parameters.

**Figure 4:**
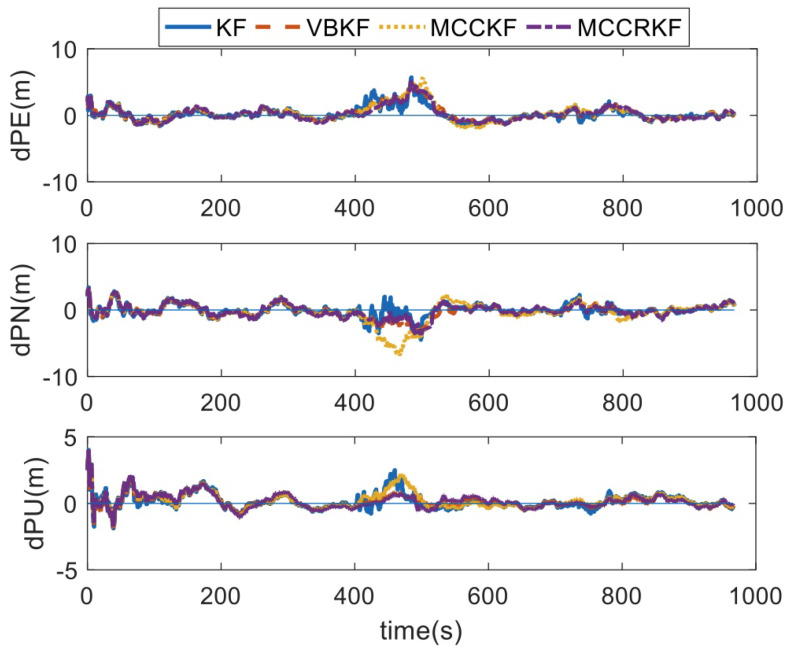
The positioning error of different algorithms under time-varying noise.

In test 3, the measurement noise distribution is shown in Equation (24), where $t_{k}$ represents the time-varying noise coefficient. The specific value is shown in Figure 3. The positioning errors of the four different algorithms are shown in Figure 4, and the RMSE errors of the positioning error statistics are shown in Table 4.

(24) $$v_{k}∼t_{k}N\left(0,R_{k}\right)$$

It can be found that, when the measurement noises are time-varying, the estimated errors of the four algorithms increase due to the degradation of GPS accuracy. However, due to the time-varying noise, the MCCKF algorithm is corrupted by these type noises, and the estimated accuracy is even lower than the same results of the Kalman filter, while the estimation errors of the VBKF algorithm and the MCCRKF algorithm have a small increase, which is within the normal range.

**Figure 5:**
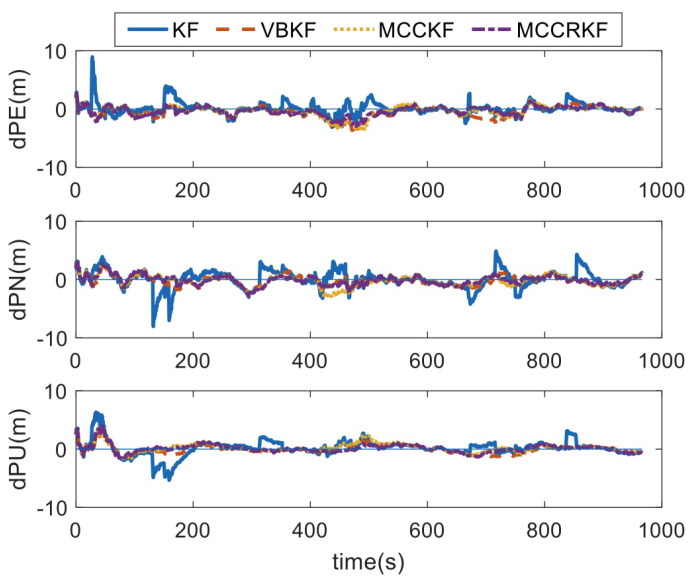
The positioning error of different algorithms under time-varying and non-Gaussian noises.

The measurement noise distribution, which is used in test 4, is shown in Formula (25). The positioning errors of the four different algorithms are shown in Figure 5, and the RMSE errors of the error statistics are shown in Table 5.

(25) $$v_{k}∼t_{k}\left(0.99N\left(0,R_{k}\right)+0.01N\left(0,400R_{k}\right)\right)$$

It can be found that the estimated errors of the MCCRKF algorithm are minimal when the noises are time-varying and non-Gaussian. And the time-varying noise is suppressed to a certain extent in the case of non-Gaussian noise suppression.

### 4.2. Field Test

To further validate the performance of the proposed method, the field test, which is carried on a vehicle in Beijing, is designed. During the test, the bias of the gyroscope is under $3^{\circ }$/h. The random walk of the gyroscope is close to $0.03^{\circ }/\sqrt{h}$, and the bias of the accelerometer is under 1000 µg. The random walk of the accelerometer is close to 100 µg/$\sqrt{\mathrm{Hz}}$. The output rates of all inertial sensors are 200 Hz. In the test, the GPS receiver, which is a product of the NovAtel Corporation, is adopted. The velocity error of the GPS receiver is around 1 m/s. The positioning error of the GPS receiver is about 2.5 m. The reference system is a high-level navigation system, which is equipped with triaxial fiber-optical gyroscopes and a triaxial quartz accelerometers. The bias of the fiber-optical gyroscopes is under $0.01^{\circ }$/h. The random walk of the fiber-optical gyroscope is close to $0.001^{\circ }/\sqrt{h}$, and the bias of the quartz accelerometer is under 100 µg. The random walk of the quartz accelerometer is close to 10 µg/$\sqrt{\mathrm{Hz}}$. The velocity error of the reference navigation is 0.01 m/s, and the position error of the reference system is 0.1 m.

**Figure 6:**
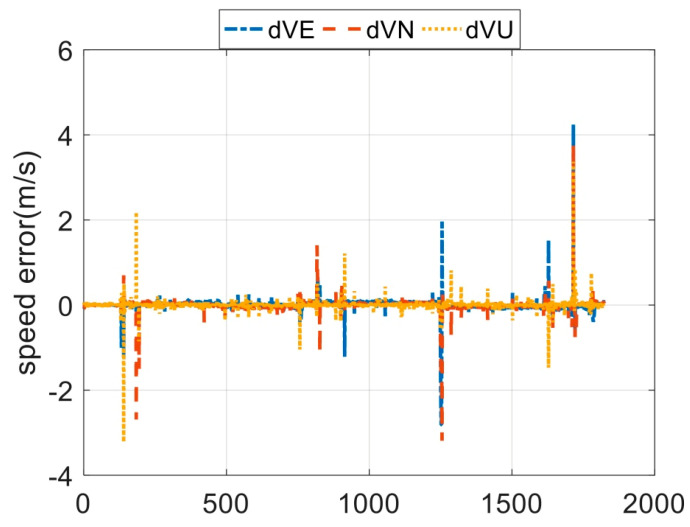
The velocity errors of the GPS outputs.

**Figure 7:**
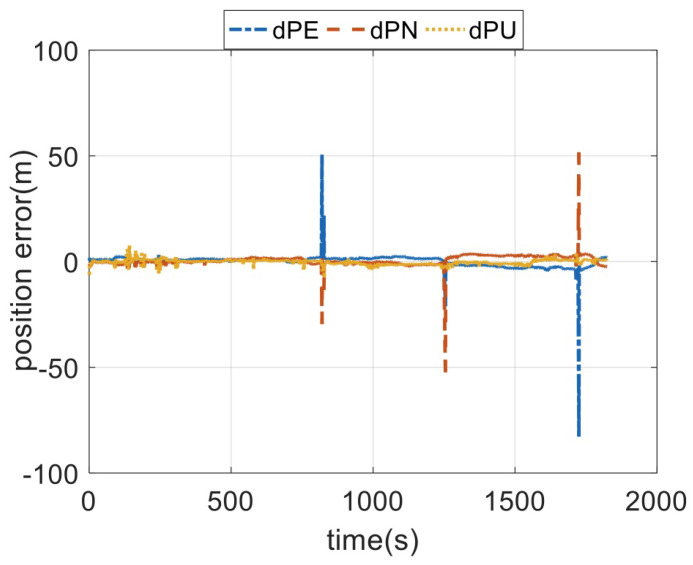
The positioning errors of the GPS outputs.

**Figure 8:**
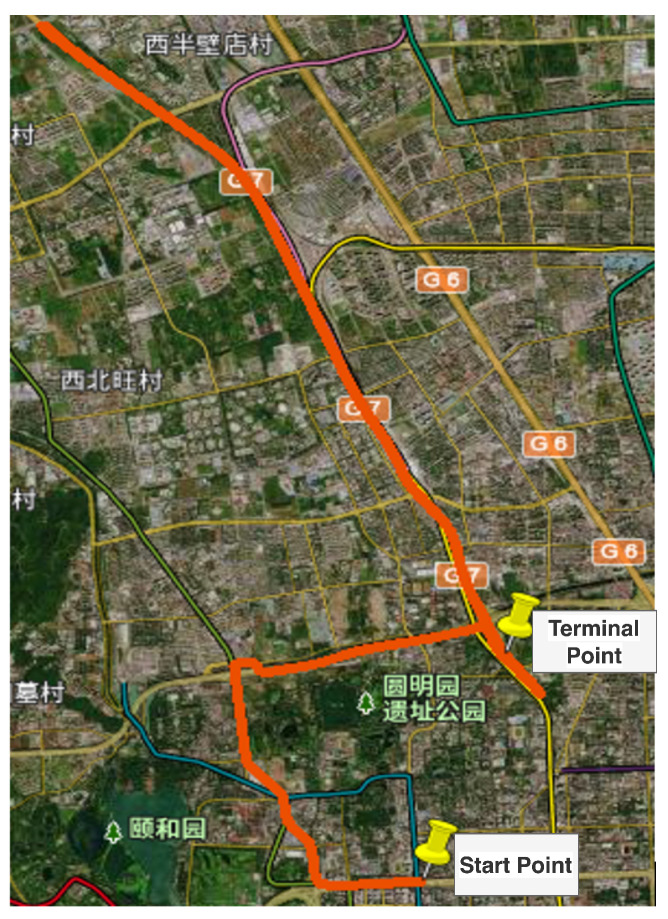
The moving trajectory of the vehicle.

The output rates of the GPS receiver is 1 Hz. The whole field test lasts for 1824 s. In Figure 6 and Figure 7, the measurement errors of the GPS receiver are shown. In Figure 6 and Figure 7, the outliers are contained. Lastly, the in-motion trajectory of the vehicle is shown in Figure 8.

**Figure 9:**
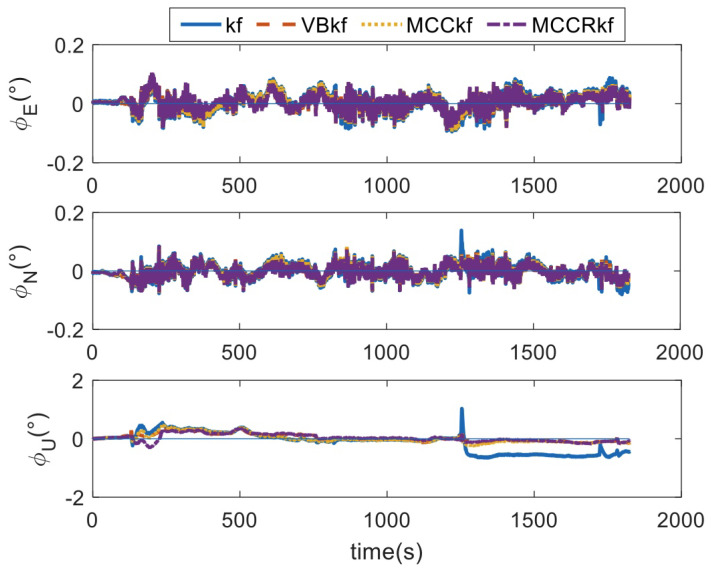
The attitude errors.

**Figure 10:**
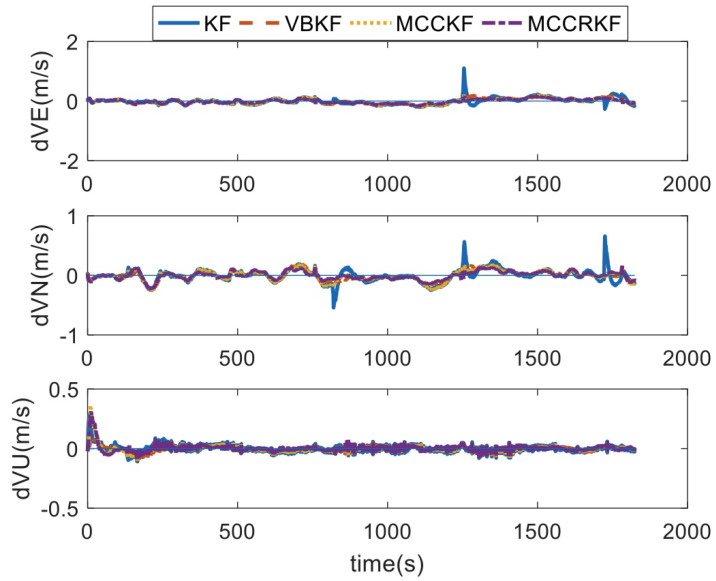
The velocity errors.

**Figure 11:**
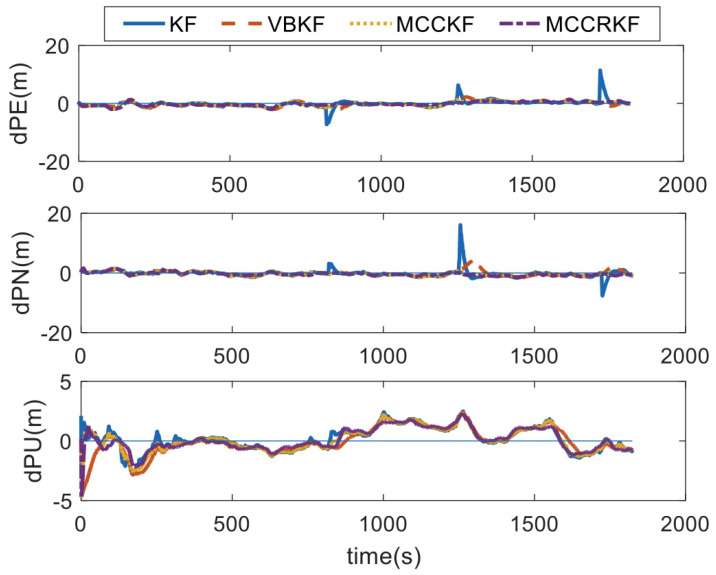
The positioning errors.

In Figure 9, Figure 10 and Figure 11, the attitude, velocity, and positioning errors of the four methods are shown, and the RMSE errors of the four methods are lists in Table 6 and Table 7. As can be seen from the attitude errors in Figure 9, the horizontal attitude errors do not diverge; this is because the horizontal attitude is constrained by the accelerometers’ measurements. However, the yaw angles diverge when the integrated system lasts for 1250 s; this is because the yaw angle is determined by the GPS measurements. When the outliers are contained in the GPS outputs, the accuracy of the yaw angle degrades.

In Figure 10, it can be found that the velocity errors converge to a small value when there are no outliers in the GPS outputs. However, when there are outliers, the number of errors of the KF method increase. With the robust filter method, the errors of VBKF, MCCKF, and MCCRKF are not corrupted by the outliers. It is noted that the up velocity is not easily corrupted by the outliers; this is because the height channel is damped in this integrated system.

As can be seen from the position error comparison diagram, the KF algorithm and VBKF algorithm diverge to different degrees when there are outliers in the GPS horizontal position, while the MCCKF algorithm and the MCCRKF algorithm are almost not affected by outliers. There is obvious time-varying noise during 130 s to 230 s of GPS celestial position, which leads to the decrease in GPS accuracy. The estimation errors of the four algorithms all increase, but the MCCRKF algorithm has the smallest increase, indicating that the MCCRKF algorithm has good adaptability and robustness.

## 5. Conclusions

In this paper, an adaptive and robust Kalman filtering algorithm based on maximum correlation entropy is proposed to solve the problem that the SINS/GPS integrated navigation system estimation accuracy decreases due to the influence of non-Gaussian and time-varying noises in complex environments. Based on the MCCKF algorithm, the decay factor was calculated by a fixed-length sliding window. Then, the R matrix was estimated by using the decay factor to suppress the time-varying noises, which are contained in the GPS outputs. The performance of the proposed algorithm is verified by simulation and field tests. The tests results show that the MCCRKF algorithm can not only keep the estimation accuracy of the SINS/GPS integrated navigation system under the condition of non-Gaussian noise but also avoid the decrease in the estimation accuracy of the SINS/GPS integrated navigation system under the condition of time-varying noise.

## Figures and Tables

**Table 1:** Experimental parameters.

Types	Error Terms	Error Value
Errors of Instruments	bias of gyroscopes	$3^{\circ }$/h
	random walk of gyroscopes	$0.03^{\circ }/\sqrt{h}$
	bias of accelerometers	1000 µg
	random walk of accelerometers	100 µg $\sqrt{\mathrm{Hz}}$
	velocity errors of GPS	1 m/s
	positioning errors of GPS	2.5 m
Initial Errors of SINS	attitude errors	$\left[60^{\prime \prime }\;60^{\prime \prime }\;60^{\prime }\right]$
	velocity errors	1 m/s
	positioning errors	2.5 m

**Table 2:** The RMSE errors of four methods.

Types	KF	VBKF	MCCKF	MCCRKF
Latitude Error (m)	0.6142	0.6156	0.6146	0.6264
Longitude Error (m)	0.6427	0.6601	0.6599	0.6524
Height Error (m)	0.5853	0.6591	0.5938	0.6251

**Table 3:** The RMSE errors of four methods.

Types	KF	VBKF	MCCKF	MCCRKF
Latitude Error (m)	1.7963	0.8570	0.7238	0.7747
Longitude Error (m)	1.1676	1.0291	0.6381	0.6260
Height Error (m)	1.1109	0.6222	0.6153	0.6188

**Table 4:** The RMSE errors of four methods.

Types	KF	VBKF	MCCKF	MCCRKF
Latitude Error (m)	0.9561	0.7685	0.6886	0.7897
Longitude Error (m)	1.1705	0.7881	1.7656	0.7795
Height Error (m)	0.7313	0.6989	0.6340	0.6941

**Table 5:** The RMSE errors of four methods.

Types	KF	VBKF	MCCKF	MCCRKF
Latitude Error (m)	1.5112	1.1097	1.2023	0.8520
Longitude Error (m)	1.2113	1.0052	0.8344	0.7199
Height Error (m)	0.9170	0.6098	0.6296	0.5890

**Table 6:** The RMSE errors of velocity.

Types	KF	VBKF	MCCKF	MCCRKF
East Velocity Error (m/s)	0.1069	0.0782	0.0842	0.0700
North Velocity Error (m/s)	0.1061	0.0762	0.0886	0.0666
Up Velocity Error (m/s)	0.0285	0.0275	0.0332	0.0309

**Table 7:** The RMSE errors of position.

Types	KF	VBKF	MCCKF	MCCRKF
East Velocity Error (m/s)	1.2671	0.7943	0.7580	0.6481
North Velocity Error (m/s)	1.3406	0.8658	0.6964	0.6254
Up Velocity Error (m/s)	0.9871	1.1223	1.0069	0.9295

## Data Availability

Not applicable.
